# Proof-of-concept of a co-designed mobile game prototype to enhance parental mentalizing: Results from a single-group pretest-posttest feasibility trial (HelpingParentsMinds)^☆^

**DOI:** 10.1016/j.invent.2026.101003

**Published:** 2026-09-29

**Authors:** L. Braun, L. Bauer, J. Engelmann, P. Rizzo, E. Merkenschlager, J. Volkert

**Affiliations:** a Clinic for Psychosomatic Medicine and Psychotherapy, Section Translational Psychotherapy, University of Ulm, Ulm, Germany; b Interagens s.r.l., Rome, Italy

**Keywords:** Mentalizing, Parenting, Internet-based intervention, Mobile health, Serious games, Co-design

## Abstract

**Background:**

Parental stress, a key risk factor for child maltreatment, is rising. The Lighthouse Parenting Program (LPP) shows promise in improving mentalizing and reducing parental burden. However, numerous barriers can hinder participation in face-to-face prevention programs. Digital interventions might help overcome barriers, with serious games considered particularly suitable for promoting intervention adherence. This study evaluated the feasibility of a mobile game prototype to support parental mentalizing and reduce stress.

**Methods:**

Feasibility was assessed in a one-group pretest-posttest study. The low-fidelity prototype was co-designed with burdened parents and based on the LPP and the Mediational Intervention for Sensitizing Caregivers. Parents are supported in reflecting on their own and their child's mental states in challenging parent-child interactions. Eligibility criteria included age ≥ 18 years, at least one child (age 0–14), an Android smartphone and sufficient language skills. Primary outcomes were recruitment, satisfaction, usability, and prototype use. Secondary outcomes include stress, depressive symptoms and mentalizing. Post-assessment followed a 10-day intervention phase.

**Results:**

Recruitment took four weeks, yielding *N* = 26, exceeding the target of *N* = 22. Usability was rated good (SUS: M = 77.76, SD = 10.96, grade B+), though satisfaction was low (CSQ-I: M = 18.74, SD = 4.47). Nineteen participants (73.1%) used the prototype for 11 min on average. Depressive symptoms and stress decreased from pre- to posttest.

**Discussion:**

Low satisfaction likely reflects the prototype's preliminary stage in this proof-of-concept study, with limitations regarding content, design, and gamification. Usability and feedback along with exploratory outcomes were promising, although feasibility has not been fully achieved. A high-fidelity version needs to be evaluated in future studies.

## Highlights

• A proof-of-concept for a mobile game to enhance parental mentalizing was developed
• A participative co-design approach with parents was used to create the prototype
• The low-fidelity prototype showed high usability in feasibility testing
• Low satisfaction was likely due to limited content, design, and gamification
• Depressive symptoms and stress decreased from pre- to post-intervention

## 1 Introduction

In the wake of global crises, a growing body of research suggests that the experience of parental stress and related mental health difficulties are on the rise. Parents of underage children experience many individual, interpersonal and organizational factors which can lead to parental stress, describing the emotional, psychological and physical strain arising from the various responsibilities of parenting (Rusu et al., 2025). This can lead to parental burnout, following chronic parental stress and being characterized by exhaustion, emotional distancing and the feeling of ineffectiveness in the parental role (Mikolajczak et al., 2018; Ren et al., 2024). Accordingly, subclinical and clinically relevant depression and anxiety symptoms increased from 15.7% in 2015 to 21.5% among parents of children younger than 3 years in 2022 in Germany (Hänelt et al., 2024). Furthermore, the Covid-19 pandemic led to an exacerbation of parental stress and specifically, in a subgroup of parents, to clinical levels of depression and anxiety, associated with an increase of verbal emotional abuse and domestic violence toward their children (Calvano et al., 2022). Importantly, parental stress needs to be understood within the broader social, economic, relational, and institutional contexts in which parenting takes place, including the availability of social support and family-oriented resources (d’Orsi et al., 2026; Nomaguchi and Milkie, 2020). Poorer socioeconomic status, for example, has been associated with higher parental stress (Martins et al., 2023). Moreover, child characteristics, such as the presence of children's behavioral, emotional, developmental or cognitive problems may also increase parental stress (d’Orsi et al., 2026). Parental stress and mental health issues are among the most common micro-level risk factors for child maltreatment (Younas and Gutman, 2023). Based on a meta-analytic review, parental stress during the COVID-19 pandemic was associated with increased internalizing and externalizing behaviors in children, poorer parental mental health, more negative parenting behaviors, and disruptions in the parent–child relationship (Chung et al., 2024). Longitudinally, higher parental stress has been shown to significantly predict a higher risk of future child maltreatment (Han et al., 2024). Moreover, mental illness of parents has been associated with impaired family functioning and psychological problems in their children (Wiegand-Grefe et al., 2019) with parental coping behavior being a protective factor for the mental health of the children (Sell et al., 2021).

Targeting parental stress through preventive measures is therefore of great importance to protect parental mental health and reduce the negative mental health impact on the children of burdened parents. Parenting programs have been shown to improve parental outcomes including mental health, parenting practices and parent-child interaction (Branco et al., 2022). In particular, the Lighthouse Parenting Programme (LPP) is a mentalization-based group treatment for parents aiming to prevent child maltreatment (Byrne et al., 2019). Parental mentalizing is considered important for sensitive caregiving and for children's developing capacity to understand their own and others' mental states from a developmental and attachment perspective (Fonagy and Target, 1997). The LPP builds on this framework, by seeking to foster parental mentalizing through the metaphors of a sea voyage, guiding parents to better understand and reflect on their children's emotions, thoughts, and intentions, as well as their own mental states (Volkert et al., 2019). The program showed promising effects regarding improvement of mentalizing, parental adjustment, family functioning along with reduced negative parenting behaviors based on a non-randomized pre-post study (Gervinskaitė-Paulaitienė et al., 2023). A recently conducted randomized controlled trial (RCT) in England compared the LPP with usual care in child protection services and found no significant differences in child abuse potential or social care status as primary outcomes. The authors concluded, based on interviews and focus groups, that more intensive training for the family support workers and social workers, who delivered the training, was needed (Sleed et al., 2025). A RCT conducted in psychiatric hospitals in Germany compared an LPP-based parenting program with psychoeducation for parents with mental illnesses and found improvements in parenting practices, parental stress, and additional exploratory outcomes (Herpertz et al., 2025). A recent RCT conducted in China found greater improvements in parenting stress after a culturally adapted LPP version compared to waitlist control group in a sample of parents with elevated stress levels (Hu et al., 2026). Moreover, an online group therapy version of the LPP implemented in primary care in Ireland showed pre-post within-group reduction of parental stress in parents with children with emotional and behavioral difficulties (Spollen et al., 2026). The LPP was developed in the UK and is already being implemented and evaluated regarding its potential to alleviate parental burden in different populations and formats across various countries including Germany (Georg et al., 2024; Volkert et al., 2019), Denmark (Hestbaek et al., 2025), Portugal (Harfouche, 2022), Lithuania (Gervinskaitė-Paulaitienė et al., 2024), Chile (Costa-Cordella et al., 2025), and Iran (Rahimi et al., 2025).

Although promising approaches such as the LPP exist to alleviate parental stress, various barriers may prevent parents from seeking and accessing mental health care. Possible obstacles reported by parents with a low socioeconomic status regarding receiving child health promotion information were time, work schedule, transportation and their own health (Davis et al., 2015). The most frequently reported barriers to participation in parenting programs from the participants' perspective in a systematic review were stigma and gender-related issues, competing demands on parents' time and resources, group dynamics, accessibility and suitability of the venue, and lifestyle factors (Mytton et al., 2014). As a further example, parents described concerns about wasting their own or the healthcare professional's time and reported fears of possible negative consequences when seeking advice from healthcare professionals regarding their children's sleep difficulties (Cook et al., 2020).

Digital mental health services offer the means of bridging this gap and provide broader reach and low-threshold accessibility to underserved groups (Lattie et al., 2022). From a stakeholders' perspective, digital mental health interventions offer several advantages, including low structural barriers due to time- and location-independent access, reduced psychological barriers through anonymity and the absence of face-to-face contact, stigma reduction facilitated by psychoeducation, and potential for personalization (Kuso et al., 2021). Digital mental health interventions can also serve as a stepping stone to access face-to-face prevention offers (Bond et al., 2023). However, numerous existing parenting apps are of low quality, frequently lacking a theoretical basis and empirical scientific validation (David et al., 2024; Davis et al., 2017; Virani et al., 2019). Moreover, for promising mobile health applications such as the user-centered developed and scientifically evaluated i-pregno app for reduction of psychosocial parental burden in the postpartum period challenges related to intervention adherence were reported (Henning et al., 2025). Limited engagement with digital mental health interventions is widely described in the literature as a common problem that diminishes their real-world impact during implementation (Lipschitz et al., 2023).

In contrast, gamification and the use of serious games are gaining attention in mental health care as alternative digital intervention formats due to their perceived potential to improve engagement (Damaševičius et al., 2023; Vindigni, 2023). Accordingly, a systematic review reported that 70% of included studies found either significant effects or positive trends of gamification on adherence (De Croon et al., 2021). Serious games are not designed solely for entertainment, but are developed to achieve educational, training, or health-related goals as their primary objective (Laamarti et al., 2014). While there are many serious games developed for children, youth or young adults and their parents targeting neurodevelopmental disorders such as autism spectrum disorder (Derks et al., 2022) or attention-deficit/hyperactivity disorder (Doulou et al., 2025) and mental disorders such as depression and anxiety (Martinez et al., 2021), there is a considerable research gap pertaining game-based digital interventions for targeting parental distress. *Happy for families* is a serious game that focuses on enhancing emotional competencies of parents, that showed promising effects on emotional awareness and regulation, alongside increased use of positive reappraisal strategies in parents (Priego-Ojeda et al., 2025). Further recent developments include the game *EmoBalance* aiming to train emotion regulation skills of parents by solving challenging situations of parent-adolescent interactions and learning different emotion regulation strategies (Pereira et al., 2024), as well as the game *Emotion Odyssey* that uses the concept of an escape room to introduce both adolescents and parents in a multi-player approach to basic emotions and emotion regulation strategies (Silva et al., 2025), both of which have not yet been evaluated with regard to their clinical effectiveness.

Therefore, our aim was to develop a serious game for burdened parents, grounded in the LPP, to foster parental mentalizing and support parents in adopting their children's perspective when reflecting on their children's emotions, thoughts, and behaviors, as well as their own. The aim of a digital LPP version was to make its content more accessible and flexible for parents who may face practical barriers to participate in a group intervention and to support engagement and adherence with the intervention by using a gamification approach. In a first step, we developed a low-fidelity game prototype in a participatory approach involving interested parents. Results from the qualitative interviews and focus groups conducted during the co-design of this game prototype are reported elsewhere (Braun et al., n.d.). In this article, we report the results of the feasibility study we conducted to evaluate the preliminary game prototype using a formative approach. The primary objective was to evaluate feasibility, acceptability, usability and engagement with this game prototype. In addition, a one-group pre–post design was used to examine exploratory clinical outcomes, to provide preliminary, formative information for intervention development and outcome selection for future evaluations.

## 2 Material and methods

### 2.1 Study design

A single-group pre-posttest trial was conducted to evaluate feasibility of a mobile game prototype. The trial is reported according to the updated CONSORT 2025 guidelines for reporting randomized trials (Hopewell et al., 2025), the extension to randomized pilot and feasibility trials (Eldridge et al., 2016) as well as the guidelines for reporting non-randomized pilot and feasibility studies (Lancaster and Thabane, 2019). The study was approved by the ethics committee at University of Ulm (No. 233/24, 03.10.2024) and preregistered on OSF (https://osf.io/sur8z/, 08.12.2024). The trial was carried out between 12/2024 and 02/2025. Online-assessments were conducted via Unipark at baseline (T0) and ten days after invitation to the test phase (T1).

### 2.2 Study sample

Recruitment took place from 12/2024 to 01/2025. Recruitment channels included advertisement through social media and distribution of leaflets and posters in counseling centers, kindergartens, youth centers, pharmacies, pediatrician practices and the university. The project was presented to patients at the Clinic for Psychosomatic Medicine and Psychotherapy at the University Hospital Ulm. Also, participants from a previous study on the co-design of the mobile game prototype (Braun et al., n.d.) were invited. Interested parents were eligible if they were (1) 18 years or older, (2) had sufficient German language skills, (3) had at least one child younger than 15 years, and (4) had access to an Android smartphone. Exclusion criteria were (1) acute suicidal ideation, (2) acute psychotic episodes and (3) intoxication or substance dependence.

### 2.3 Procedure

Interested parents were invited to an online-screening. Eligible parents provided informed consent and were invited to the baseline survey (T0). Upon completion, participants received instructions on how to access the mobile game prototype for the test phase. The participants received a personalized download link to test the prototype on their own device (BYOD, Bring Your Own Device) after they created a pseudonymized study mail address. Participants were not given any instructions on the frequency and duration of prototype use during the test phase. Ten days after receiving the download link, participants were invited to the post survey (T1) and compensated with EUR 40.

A safety procedure was implemented for the assessment of suicidal ideation using the suicidality item of the PHQ-9 (“Thoughts that you would be better off dead or of harming yourself”). Participants who responded with ‘several days’ (1) were advised to contact clinical staff if they were currently inpatients at our psychosomatic clinic and provided with information on support services and crisis contacts. Participants who responded ≥1 were further advised to immediately contact one of the listed emergency services if they experienced specific suicidal thoughts and felt at risk of acting on these thoughts.

### 2.4 Intervention

A low-fidelity prototype for a mobile game has been developed in a participative multi-step co-design process including team discussions among the involved researchers, as well as qualitative interviews and focus groups with interested parents. The experiential expertise of parents regarding difficult everyday situations with the child, as well as their needs and expectations were considered in designing the framework of the game prototype. The co-design process is described in detail elsewhere (Braun et al., n.d.). The feasibility testing of this preliminary, low-fidelity version of a mobile game is one step in the co-design development of a sophisticated mobile application to support parental mentalizing. Low-fidelity prototyping can be similarly effective as high-fidelity prototyping in revealing usability problems (Virzi et al., 1996). Formative iterative evaluations throughout the design, development, and deployment of eHealth interventions, as opposed to standalone randomized controlled trials conducted only at the end of the development process, are beneficial for incorporating contextual considerations and maximizing the potential impact of eHealth interventions (Catwell and Sheikh, 2009). Technical development of the low-fidelity prototype was conducted by the IT development partner Interagens (http://www.interagens.com/) on the Firebase platform (https://firebase.google.com/).

The concept of the game prototype is based on the Lighthouse Parenting Programme (LPP) (Byrne et al., 2019) as well as the Mediational Intervention for Sensitizing Caregivers (MISC) (Sharp et al., 2020). The LPP is a parenting program based on the principles of Mentalization-Based Therapy (MBT) (Bateman et al., 2023). The program is built around the metaphors of a sea voyage, with the parents symbolized by the lighthouse. The parents' momentary mentalizing ability, i.e. the capacity to reflect on their own thoughts, feelings, and behaviors, as well as those of their child, is represented by the illuminating beam transmitted by the lighthouse, which may vary in intensity. The metaphor of the safe harbor describes the ability of the parents to provide a safe space for the child. Further metaphors represent parent-child interactions, for example “the raft”, when the child seems distanced and is hard to be reached, or “piracy”, when the child is angry and hard to soothe. The parents are encouraged throughout the program to switch on their illuminating beam, i.e. to mentalize both themselves and their children, and guide them back to the safe harbor. The prototype as shown in Fig. 1 is centered around the LPP integrating the different metaphors and images. Parents are able to select challenging parent-child interactions (1). Based on multiple-choice questions, parents are instructed to reflect about their own mental states (2), to calm themselves as a prerequisite to being able to mentalize and to gain a deeper understanding of the mental states of their child (3). This is followed by the explanation of the accompanying Lighthouse metaphor and psychoeducational information (4–5). To provide practical guidance how to reconnect with the child the MISC model is applied. The MISC model provides five mentalization-based steps, to create a shared focus on the situation (step 1: focusing), provide meaning to the situation (step 2: affecting), consider the situation in its broader context (step 3: expanding), validate the child's behavior (step 4: rewarding), and show the child how to plan ahead (step 5: regulating). For each step of the MISC model, the prototype presents various response options, enabling parents to practice different strategies and evaluate their impact on their relationship quality (6–7). When parents choose unhelpful options, they are encouraged to reconsider and select an alternative strategy (8). When parents achieve a positive outcome and the child returns to the safe harbor, they receive a congratulatory message as positive reinforcement. The parents are then asked if they are in a similar situation and would like to try the strategies they have learned in real life (9). If so, the app provides brief reminders and switches to stand-by mode during parent-child interaction. Afterwards, parents are asked to rate their satisfaction with their handling of the situation and to indicate their current level of stress (10). Overall, the preliminary prototype we tested in this feasibility study for proof-of-concept included two different exemplary situations for difficult parent-child interactions for children of different ages.

**Fig. 1:**
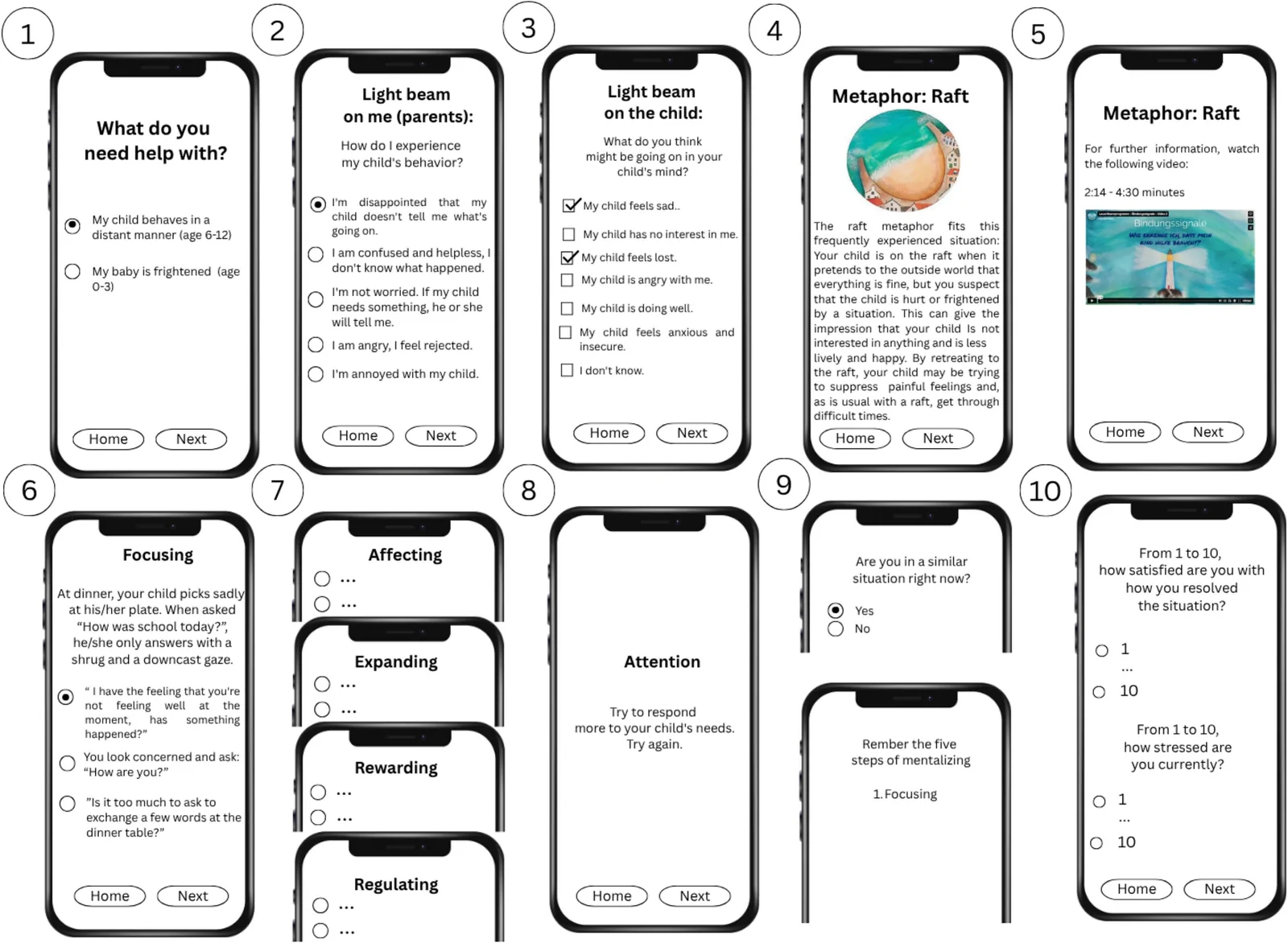
Exemplary presentation of the mobile game prototype structure. Note. 1) Situation selection; 2) Reflection of own feelings (mentalizing oneself); 3) Reflection of child's mental states (mentalizing the child); 4) Explanation of the corresponding metaphor from the LPP; 5) Link to psychoeducational videos (available online: https://www.leuchtturm-elternprogramm.de/videos); 6) Step 1 of the MISC model (Focusing): Parents can select different response options in the simulated situation; 7) Step 2–5 of the MISC model with different response options for each step; 8) Reflective prompt triggered by selection of non-mentalizing responses; 9) Support transfer to real-life situations; 10) Evaluation of satisfaction and momentary stress.

### 2.5 Outcome measures

#### 2.5.1 Feasibility measures: Recruitment, intervention satisfaction, usability, intervention initiation and engagement

Feasibility was assessed across several complementary dimensions. Recruitment strategies were reported to provide information on the feasibility of recruiting participants for future evaluations. Different recruitment strategies were compared in terms of recruitment success. Sociodemographic characteristics (e.g. age, gender, education level, socioeconomic status) were assessed to characterize the population reached via the recruitment channels applied. Intervention satisfaction was included as an indicator of acceptability, whereas usability assessed participants perceived ease and appropriateness of interacting with the prototype. Intervention satisfaction was assessed with the Client Satisfaction Questionnaire for Internet Interventions (CSQ-I) on a four-point Likert scale with a total score between 8 and 32 (Boß et al., 2016) with high reliability (ω_t1_ = 0.84). Based on multicenter data from inpatient rehabilitation a satisfaction score of >23 can be classified as good in psychosomatic patients (Kriz et al., 2008). Usability of the prototype was measured using the System Usability Scale (SUS) (Brooke, 1996). Ten items were rated on a five-point Likert scale and calculated to a SUS score ranging from 0 to 100 (Brooke, 1996) with sufficient internal consistency (ω_t1_ = 0.69). Although the procedure used to establish pseudonymized access to the prototype represented a pragmatic solution for the present study rather than a predefined feasibility outcome, the number of participants that did not complete this process provide relevant information on potential implementation barriers. Frequency of prototype use and average engagement time were included as behavioral indicators of engagement with the intervention. Intervention use was assessed based on self-report of participants regarding frequency and duration of use. In addition, objective use data was collected via the Firebase platform using its standard engagement metrics. Average engagement time refers to the time during which the app was in the foreground on the user's device. An engaged session is defined as a session lasting at least 10s, including at least one key event, or including at least two screen views. We report the sample average engagement time, the sample average number of sessions engaged, and the sample average engagement time per session as behavioral indicators of app use. Finally, two open-ended feedback questions were included to assess positive features as well as potential barriers (1: “What did you like about the prototype?” 2: “What could be improved, what is missing, what would you have liked instead?”). Together, these measures were intended to capture key aspects of feasibility, including recruitment, acceptability, usability, intervention initiation and engagement, and to identify aspects requiring optimization before subsequent evaluation.

#### 2.5.2 Preliminary clinical outcome measures

The Parenting Stress Index (PSI) was used to assess both child- and parent-related sources of burden (Tröster, 2011) with excellent reliability (ω_t0_ = 0.96, ω_t1_ = 0.96). The Perceived Stress Scale (PSS) was used to assess parental stress (Cohen et al., 1983) with good internal consistency (ω_t0_ = 0.91, ω_t1_ = 0.85). The German version of the Patient Health Questionnaire (PHQ-D) was applied to screen for stress, alcohol abuse, somatization, depressive, panic, anxiety and eating syndromes (Gräfe et al., 2004). The PHQ-D entails the PHQ-9 for screening of depressive symptoms (ω_t0_ = 0.85, ω_t1_ = 0.76), the PHQ-15 for assessing somatoform symptoms (ω_t0_ = 0.84, ω_t1_ = 71) and the PHQ-stress for evaluating stress (ω_t0_ = 0.83, ω_t1_ = 0.79). The Parental Reflective Functioning Questionnaire (PRFQ) was used to measure parental mentalizing based on three dimensions (Luyten et al., 2017) with sufficient reliability. Pre-mentalizing (PM: ω_t0_ = 0.73, ω_t1_ = 0.77) describes a non-mentalizing attitude, certainty about mental states (CMS: ω_t0_ = 0.83, ω_t1_ = 0.85) includes either being overly certain about children's inner states or having no certainty at all, and interest and curiosity (IC: ω_t0_ = 0.54, ω_t1_ = 0.70) means either an absence of interest or an intrusive way of mentalizing. Emotion regulation deficits were assessed with the short version of the Difficulties in Emotion Regulation Scale (DERS-SF) (Gratz and Roemer, 2004) with overall excellent reliability (ω_t0_ = 0.98, ω_t1_ = 0.97). The six subscales of emotion dysregulation (1) strategies (ω_t0_ = 0.83, ω_t1_ = 0.86), (2) nonacceptance (ω_t0_ = 0.88, ω_t1_ = 0.96), (3) impulse (ω_t0_ = 0.88, ω_t1_ = 0.42), (4) goals (ω_t0_ = 0.92; ω_t1_ = 0.92), (5) awareness (ω_t0_ = 0.66; ω_t1_ = 0.77), and (6) clarity (ω_t0_ = 0.87; ω_t1_ = 0.82) were found to have sufficient internal consistencies. Finally, the relationship quality was assessed with the multi-perspective questionnaire on the parent-child relationship (MEK) with the two subscales emotional bond (ω_t0_ = 0.72, ω_t1_ = 0.67) and functional conflict (ω_t0_ = 0.63, ω_t1_ = 0.78) (Müller and Achtergarde, 2018).

### 2.6 Statistical analyses

A sample size of 18.4 was calculated following Viechtbauer et al. (2015) (http://www.pilotsamplesize.com) based on an assumed probability of 15% of encountering feasibility problems and a confidence level of 95%. A meta-analysis examining internet-based parenting programs found dropout rates between 7% and 40% (Florean et al., 2020). Assuming an average dropout rate of 20%, the study sample size was set a priori to *N* = 22.

Data analysis was conducted in R 4.4.2 (R Core Team, 2020). All analyses are based on a significance level of *p* < .05. Normality and homogeneity of variances were tested using Shapiro-Wilk and Levene's tests. Descriptive analyses (M, SD) were reported for the feasibility outcomes. Spearman's rho correlations were used to examine associations between feasibility outcomes, usage, and psychological burden. Open-ended questions were analyzed by forming categories and calculating their frequency.

For preliminary clinical outcomes, *t*-tests for paired samples (two-sided) were applied. In cases of non-normality, Wilcoxon signed-rank tests were performed additionally. Effect sizes are reported based on Cohen's d. As pre-post analyses were of highly explorative nature, no Bonferroni correction for multiple comparisons was applied (Bender and Lange, 2001). Participants who failed to complete intervention enrollment were not invited to the post-assessment and were classified as non-initiators. Pre-post analyses were conducted among intervention initiators to describe within-person changes over time, rather than to estimate intervention efficacy, given the formative feasibility design.

Baseline comparisons between initiators and non-initiators were conducted using two-sided independent *t*-tests following checks for normality. For categorial variables, Fisher tests (*n* < 5 per cell) were used. Internal consistencies of the selected measures were calculated using McDonald's omega, which provides a robust estimate of reliability (Hayes and Coutts, 2020).

## 3 Results

### 3.1 Feasibility measures: Recruitment, intervention satisfaction, usability, initiation and engagement

#### 3.1.1 Recruitment and intervention initiation

Different recruitment strategies were applied as shown in Table 1. Most participants were recruited from the previous co-design study (*n* = 8, 26.7%), through personal contacts or distributed leaflets and posters (*n* = 7, 23.3, each) and through clinical staff *n* = 6 (20.0%). The study flow is shown in Fig. 2. In total, *N* = 90 persons accessed the screening. 30 eligible participants completed the screening and gave informed consent for study participation. A total of *N* = 26 participants completed T0 and were included in the study, exceeding the a priori defined sample size of *N* = 22 because additional participants registered before the survey was closed. 7 (26.9%) participants failed to create a pseudonymized study email address for the test phase invitation and did not initiate the intervention. All *N* = 19 participants, who initiated the intervention, completed the post-survey (T1) after the test phase.

**Table 1:** Recruitment strategies.

Strategies	*N (%)* recruited
Invitation of participants from previous co-design study	8 (26.7)
Distribution of leaflets and posters (e.g. kindergardens, schools, pediatricians, pharmacies, children's theater, university)	7 (23.3)
Recruitment through clinical staff	6 (20.0)
Recruitment through outpatient psychotherapists	1 (3.3)
Recruitment through personal contacts (e.g. wife, colleague, friends)	7 (23.3)
Press release	1 (3.3)
Emails sent to different recipients (e.g. counseling centers, parent's councils, child protection agencies)	0 (0)
Social media and website posts	0 (0)

*Note. N* = 30 participants completing the screening. The number of recruited participants for each strategy was approximated based on information from the screening process and additional recruitment channels.

**Fig. 2:**
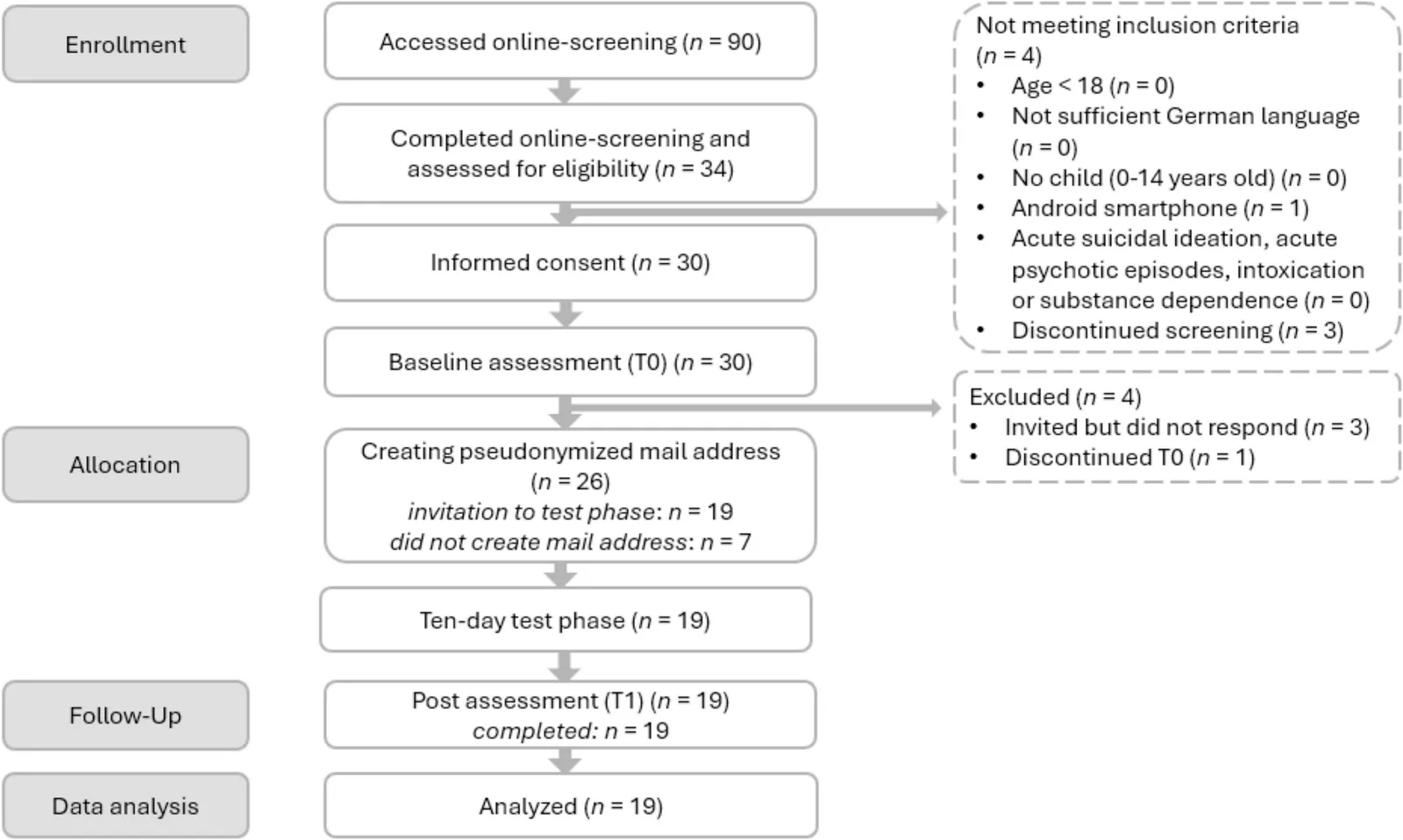
CONSORT flow chart.

Participants were on average 35.7 (SD = 5.2) years old with most participants being mothers (*n* = 19, 73.1%), married (*n* = 19, 73.1%), with middle (*n* = 8, 30.8%) to high (*n* = 15, 57.7%) educational level and being part-time employed (*n* = 12, 46.2%). The majority had two children (*n* = 19, 73.1%), with an average child age of 4.0 (SD = 3.1) years. Most of the participants reported minimal to mild levels of depressive symptoms (*n* = 18, 69.2%) and somatization (*n* = 19, 73.1%) at T0. *N* = 8 (30.8) reported a previous mental disorder with 5 (19.2%) participants having been in psychological treatment. Non-initiators differed in household income from initiators (*p* = .041), and received more often psychological treatment in the past (*p* = .010). Details on sociodemographic characteristics, parental burden, stress, and mental health symptoms are presented in Table 2 and Table 3.

**Table 2:** Sociodemographic participant characteristics.

Variable		Total (*N* = 26)	Initiator (*n* = 19)	Non-initiator (*n* = 7)	*p*^d^
Age (*M, SD*)		35.7 (5.2)	35.2 (5.7)	37.1 (3.6)	0.399
Age of children (*M, SD*)		4.0 (3.1)	3.6 (2.3)	5.1 (4.7)	0.292
Number of children					0.788
	1 child, *n* (%)	4 (15.4)	3 (15.8)	1 (14.3)	
	2 children, *n* (%)	19 (73.1)	13 (68.4)	6 (85.7)	
	3 children, *n* (%)	3 (11.5)	3 (15.8)	0 (0.0)	
Gender					0.487
	Female, *n* (%)	19 (73.1)	15 (78.9)	4 (57.1)	
	Male, *n* (%)	6 (23.1)	3 (15.8)	3 (42.9)	
	Non-binary, *n* (%)	1 (3.8)	1 (5.3)	0 (0.0)	
Nationality					
	German, *n* (%)	26 (100)	19 (100)	7 (100)	
Ethnicity					
	White, *n* (%)	26 (100)	19 (100)	7 (100)	
Mother tongue					0.269
	German, *n* (%)	25 (96.2)	19 (100)	6 (85.7)	
	Other, *n* (%)	1 (3.8)	0 (0)	1 (14.3)	
Marital status					0.664
	Married, *n* (%)	19 (73.1)	13 (68.4)	6 (85.7)	
	Partnership, living together, *n* (%)	3 (11.5)	3 (15.8)	0 (0)	
	Single, *n* (%)	2 (7.7)	2 (10.5)	0 (0)	
	Partnership, living separately, *n* (%)	2 (7.7)	1 (5.3)	1 (14.3)	
Education level^a^					0.105
	Low, *n* (%)	3 (11.5)	1 (5.3)	2 (28.6)	
	Middle, *n* (%)	8 (30.8)	5 (26.3)	3 (42.9)	
	High, *n* (%)	15 (57.7)	13 (68.4)	2 (28.6)	
Household income^b^					**0.041**
	< EUR 10,000, *n* (%)	3 (11.5)	2 (10.5)	1 (14.3)	
	EUR 25,000–49,999, *n* (%)	4 (15.4)	2 (10.5)	2 (28.6)	
	EUR 50,000–74,999, *n* (%)	5 (19.2)	5 (26.3)	0 (0)	
	EUR 75,000–99,999, *n* (%)	6 (23.1)	6 (31.6)	0 (0)	
	EUR 100,000–149,999, *n* (%)	2 (7.7)	1 (5.3)	1 (14.3)	
	> EUR 200,000, *n* (%)	2 (7.7)	0 (0)	2 (28.6)	
	Not specified, *n* (%)	4 (15.4)	3 (15.8)	1 (14.3)	
Employment status^c^					0.887
	Full-time, *n* (%)	5 (19.2)	3 (15.8)	2 (28.6)	
	Part-time, *n* (%)	12 (46.2)	9 (47.4)	3 (42.9)	
	Marginal part-time job, *n* (%)	2 (7.7)	2 (10.5)	0 (0)	
	Student, *n* (%)	2 (7.7)	2 (10.5)	0 (0)	
	No job, *n* (%)	5 (19.2)	3 (15.8)	2 (28.6)	
Living situation					1.0
	Home ownership, *n* (%)	14 (53.8)	10 (52.6)	4 (57.1)	
	Rental apartment, *n* (%)	12 (46.2)	9 (47.4)	3 (42.9)	

Note.

a following International Standard Classification of Education (OECD et al., 2015): low = primary education or lower secondary education (ISCED 0–2); middle = upper secondary education, vocational training, and post-secondary non-tertiary education (ISCED 3–5); high = tertiary education degree (Bachelor's, Master's, Diploma or Doctorate) (ISCED 6–8).

b Total household income before taxes in the last 12 months.

c multiple answers allowed.

d based on *t*-test for continuous variables and Fisher's exact test for categorical variables.

**Table 3:** Psychological burden, stress, and mental health symptoms at baseline.

		Total (*N* = 26)	Initiator (*n* = 19)	Non-initiator (*n* = 7)	*p*^d^
Self-reported burden a (*M, SD*)		6.15 (2.63)	5.84 (2.48)	7.00 (3.06)	0.392
PSI					
	Parent-related (*M, SD*)	72.92 (19.59)	71.95 (17.19)	75.57 (26.49)	0.745
	Child-related (*M, SD*)	57.46 (16.34)	57.84 (15.49)	56.43 (19.77)	0.868
	*Total score* (*M, SD*)	130.38 (32.93)	129.79 (29.46)	132.00 (43.71)	0.905
PSS-10					
	Helplessness (*M, SD*)	11.42 (5.12)	11.37 (5.11)	11.57 (5.56)	0.935
	Self-efficacy (*M, SD*)	7.77 (2.61)	7.68 (2.75)	8.00 (2.38)	0.779
	*Total score* (*M, SD*)	19.19 (7.00)	19.05 (7.21)	19.57 (6.90)	0.870
PHQ-D					
	PHQ-9 (*M, SD*)	8.27 (5.65)	6.89 (4.33)	12.00 (7.39)	0.126
	PHQ-15 (*M, SD*)	8.62 (5.05)	7.58 (4.00)	11.43 (6.75)	0.196
	PHQ-stress (*M, SD*)	6.15 (4.24)	5.37 (3.61)	8.29 (5.35)	0.218
PHQ- 9 severity b					0.323
	Minimal, *n* (%)	8 (30.8)	7 (36.8)	1 (14.3)	
	Mild, *n* (%)	10 (38.5)	8 (42.1)	2 (28.6)	
	Moderate, *n* (%)	5 (19.2)	3 (15.8)	2 (28.6)	
	Moderately severe, *n* (%)	2 (7.7)	1 (5.3)	1 (14.3)	
	Severe, *n* (%)	1 (3.8)	0 (0)	1 (14.3)	
PHQ-15 severity c					0.541
	Minimal, *n* (%)	3 (11.5)	3 (15.8)	0 (0)	
	Low,*n* (%)	16 (61.5)	12 (63.2)	4 (57.1)	
	Medium, *n* (%)	5 (19.2)	3 (15.8)	2 (28.6)	
	High, *n* (%)	2 (7.7)	1 (5.3)	1 (14.3)	
PHQ-syndromes					
	Panic syndrome, *n* (%)	2 (7.7)	0 (0.0)	2 (28.6)	1^e^
	Anxiety syndromes, *n* (%)	1 (3.8)	0 (0.0)	1 (14.3)	1^e^
	Bulimia nervosa, *n* (%)	0 (0)	0 (0.0)	0 (0.0)	1^e^
	Binge-Eating, *n* (%)	2 (7.7)	0 (0.0)	2 (28.6)	1^e^
	Alcohol syndrome, *n* (%)	3 (11.5)	2 (10.5)	1 (14.3)	1^e^
Psychological disorder					0.149
	No, *n* (%)	18 (69.2)	15 (78.9)	3 (42.9)	
	Yes, *n* (%)	8 (30.8)	4 (21.1)	4 (57.1)	
Psychological treatment					**0.010**
	No, *n* (%)	21 (80.8)	18 (94.7)	3 (42.9)	
	Yes, *n* (%)	5 (19.2)	1 (5.3)	4 (57.1)	

*Note.* PSI = Parental Stress Index sum scores (range 48–240), PSS-10 = Perceived Stress Scale sum scores (range 0–40); PHQ-D = Patient-Health-Questionnaire sum scores (PHQ-9: range 0–27, PHQ-15 range 0–30, PHQ-stress: range 0–20),

a Self-reported parental burden: “How much psychological burden do you currently feel?” (1 = not burdened at all to 10 = very heavily burdened).

b Grouped by sum score: <5 = minimal; 5–10 = mild, 10–14 = moderate, 15–19 moderately severe, 20–27 = severe.

c Grouped by sum score: <5 = minimal; 5–10 = low, 10–14 = medium, > 15 = high.

d based on *t*-test for continuous variables, based on Fisher's exact test for categorical variables.

e not interpretable due to small sample size.

#### 3.1.2 Intervention satisfaction, usability, and engagement

Intervention satisfaction measured with the CSQ-I was on average low with M = 18.74 (SD = 4.47). The detailed responses are presented in Table 4. Based on the SUS score, usability was rated as good (Bangor et al., 2009) and corresponded to a Grade B+ (Lewis and Sauro, 2018) with M = 77.76 (SD = 10.96). A boxplot from the web-based analysis toolkit from Blattgerste et al. (2022) is presented in *Fig. A.1* in Appendix A. All 19 participants invited reported having installed and tested the prototype. Most participants reported a use frequency of two or three (*n* = 7, 36.8%), or three or four times (*n* = 7, 36.8%). 3 participants (15.8%) used the prototype only once, 1 daily (5.3%) and 1 multiple time a day (5.3%). The majority of participants reported that they used the prototype for a duration of 40 to 60 (*n* = 6, 31.6%) or 20 to 40 min (*n* = 5, 26.3%). Three participants (15.8%) reported that they used the prototype for 10–20 min, while another three (15.8%) used it for one to two hours. One participant (5.3%) used the prototype for less than ten minutes and one (5.3%) more than four hours. The objective data provided by the Firebase platform showed an average engagement time per user of about 11 min. This excludes the time spent on watching the external LPP videos linked by the prototype. On average, participants used the prototype 2.7 times, with each use lasting about 4 min. There was a significant moderate correlation between the duration and the CSQ sum score (ρ = 0.49, *p* = .03), a high correlation between the CSQ sum score and the SUS score (ρ = 0.54, *p* = .02), and a moderate negative correlation between the duration and the PHQ-9 sum score (ρ = −0.49, *p* = .03). For details see Appendix B.

**Table 4:** Intervention satisfaction with the game prototype.

CSQ-I item	Strongly disagree (1), *n* (%)	Somewhat disagree (2), *n* (%)	Somewhat agree (3), *n* (%)	Strongly agree (4), *n* (%)
1. The game prototype I attended was of high quality.	1 (5.3)	8 (42.1)	10 (52.6)	0 (0.0)
2. I received the kind of game prototype I wanted.	3 (15.8)	6 (31.6)	8 (42.1)	2 (10.5)
3. The game prototype has met my needs.	3 (15.8)	5 (26.3)	10 (52.6)	1 (5.3)
4. I would recommend this game prototype to a friend if he or she needs similar help.	3 (15.8)	8 (42.1)	8 (42.1)	0 (0.0)
5. I am satisfied with the amount of help I received through the game prototype.	6 (31.6)	7 (36.8)	5 (26.3)	1 (5.3)
6. The game prototype helped me deal with my problems more effectively.	5 (26.3)	7 (36.8)	7 (36.8)	0 (0.0)
7. In an overall, general sense, I am satisfied with the game prototype.	2 (10.5)	6 (31.6)	9 (47.4)	2 (10.5)
8. I would come back to such game prototype if I were to seek help again.	5 (26.3)	4 (21.1)	9 (47.4)	1 (5.3)

*Note*. Based on the Client Satisfaction Questionnaire adapted to Internet-based interventions (CSQ-I).

At baseline, four participants reported having thoughts of being better off dead or of self-harm on some days, and one participant reported experiencing such thoughts nearly every day during the past two weeks. At post-assessment, no participant reported these thoughts during the preceding two weeks which included the intervention period.

Responses to the open-ended questions revealed positive feedback from *n* = 7 participants (36.8%), who found the prototype comprehensible, simple, and easy to use, and from *n* = 6 (31.6%) participants, who appreciated the metaphors and videos provided in the LPP. In contrast, *n* = 11 participants (57.9%) expressed criticism regarding the limited selection of situations and response options, particularly the lack of representation for their children's age groups. Negative feedback related to visualization was expressed by five participants (26.3%) who criticized the amount of text and emphasized the wish for additional videos, images and animations. A detailed overview is included in Appendix C.

### 3.2 Preliminary clinical outcomes

Perceived stress showed a mean reduction of 2.89 points from pre- to posttest (95%-CI: [0.22; 5.57]), with the helplessness subscale showing a mean reduction of 2.32 points (95%-CI: [0.35; 4.28]). Depressive symptoms showed a similar pattern, with a mean reduction of 1.63 points from pre- to posttest (95%-CI: [0.30; 2.96]). Full statistical results are presented in Table 5.

**Table 5:** Means, standard deviations and within-group changes for clinical outcomes in intervention initiators (N = 19).

Outcome	T0 (M, SD)	T1 (M, SD)	Mean change (95%-CI)	t	df	*p*	Cohens d (95%-CI)
Parental stress (PSI)							
Parent-related	71.95 (17.19)	73.11 (18.74)	−1.16 [−6.00; 3.68]	−0.50	18	0.622	−0.06 [−0.32; 0.19]
Child-related	57.84 (15.49)	58.53 (15.35)	−0.68 [−3.43; 2.06]	−0.52	18	0.607	−0.04 [−0.22; 0.13]
*Total score*	129.79 (29.46)	131.63 (31.56)	−1.84 [−8.16; 4.47]	−0.61	18	0.548	−0.06 [−0.26; 0.14]
Perceived stress (PSS-10)							
Helplessness^a^	11.37 (5.11)	9.05 (4.24)	2.32 [0.35; 4.28]	2.47	18	**0.024**	0.49 [0.06; 0.91]
Self-efficacy	7.68 (2.75)	7.11 (2.49)	0.58 [−0.62; 1.77]	1.02	18	0.322	0.22 [−0.22; 0.66]
*Total score*	19.05 (7.21)	16.16 (5.34)	2.89 [0.22; 5.57]	2.27	18	**0.035**	0.44 [0.03; 0.85]
Mental health (PHQ-D)							
Depressive symptoms (PHQ-9)^a^	6.89 (4.33)	5.26 (3.93)	1.63 [0.30; 2.96]	2.58	18	**0.019**	0.39 [0.07; 0.71]
Somatization (PHQ-15)	7.58 (4.00)	6.84 (4.00)	0.74 [−0.24; 1.71]	1.59	18	0.130	0.18 [−0.05; 0.42]
Stress (PHQ-stress)	5.37 (3.61)	5.05 (3.46)	0.32 [−0.50; 1.14]	0.81	18	0.429	0.09 [−0.13; 0.31]
Parental reflective functioning (PRFQ)							
Pre-Mentalizing	2.11 (0.95)	1.96 (0.87)	0.15 [−0.13; 0.42]	1.14	18	0.268	0.16 [−0.13; 0.45]
Certainty about mental states	3.51 (1.09)	3.52 (1.08)	−0.01 [−0.32; 0.31]	−0.06	18	0.954	−0.01 [−0.29; 0.27]
Interest and curiosity	5.19 (0.79)	5.44 (0.75)	−0.25 [−0.51; 0.02]	−1.93	18	0.069	−0.32 [−0.66; 0.02]
Strategies	2.00 (0.67)	1.93 (0.93)	0.07 [−0.30; 0.44]	0.40	18	0.691	0.08 [−0.34; 0.51]
Non-acceptance	2.32 (1.10)	2.3 (1.29)	0.02 [−0.22; 0.26]	0.15	18	0.881	0.01 [−0.17; 0.20]
Impulse	1.63 (0.71)	1.54 (0.40)	0.09 [−0.20; 0.38]	0.63	18	0.537	0.14 [−0.32; 0.60]
Goals	2.44 (1.11)	2.44 (1.18)	<0.001 [−0.35; 0.35]	<0.001	18	1.00	0.00 [−0.29; 0.29]
Awareness^a^	2.61 (0.86)	2.81 (0.90)	−0.19 [−0.42; 0.03]	−1.82	18	0.086	0.22 [−0.46; 0.03]
Clarity^a^	2.11 (1.23)	1.91 (0.89)	0.19 [−0.29; 0.68]	0.83	18	0.415	0.17 [−0.25; 0.59]
*Total score*	2.18 (0.65)	2.15 (0.67)	0.03 [−0.16; 0.22]	0.32	18	0.750	0.04 [−0.23; 0.32]
Parent-child relationship (MEK)							
Emotional bond	3.38 (0.48)	3.42 (0.41)	−0.05 [−0.20; 0.11]	−0.60	18	0.555	0.10 [−0.44; 0.24]
Functional-conflict	2.99 (0.66)	3.02 (0.63)	−0.03 [−0.21; 0.15]	−0.35	18	0.732	−0.05 [−0.32; 0.22]

Note. *p*-values: two-sided paired *t*-test; significant level: *p* < .05; PSI = Parental Stress Index (range 48–240); PSS-10 = Perceived Stress Scale (range 0–40); PHQ-D = Patient-Health-Questionnaire (PHQ-9: range 0–27, PHQ-15 range 0–30, PHQ-stress: range 0–20); PRFQ = Parental Reflective Functioning Questionnaire (1–7;); DERS-SF = Difficulties in Emotion Regulation (1–5); MEK = Multi-perspective questionnaire on parent-child relationship (0–4).

a Due to violations of the normality assumption of the difference scores between T0 and T1, Wilcoxon signed-rank tests were additionally conducted, which confirmed the results of the parametric analyses.

## 4 Discussion

In this one-group pretest-posttest trial, we evaluated the feasibility of a preliminary mobile game prototype developed in a participative co-design process with burdened parents. The recruitment was satisfactory with the recruitment goal being reached after approximately four weeks. However, the reach was limited to white, German participants, with mothers comprising the majority of the sample (73.1%) and a high proportion having an academic level of education (57.7%). Based on self-reported data, prototype use ranged from a single use to multiple daily uses, with most participants reporting a use between two and four times. Usage frequency was reported to vary between less than ten minutes and more than four hours with most participants reporting a usage time between 20 and 60 min overall. In contrast, data from the Firebase platform showed an average engagement time per user of about 11 min. Average intervention satisfaction was below (M = 18.74, SD = 4.47) the recommended cut-off of a good satisfaction score (>23) in psychosomatic patients (Kriz et al., 2008). However, usability of the prototype was rated as good (Bangor et al., 2009) corresponding to a grade of B+ (Lewis and Sauro, 2018). Preliminary within-group changes from pre- to posttest were found for depressive symptoms and perceived stress.

The findings suggest that this study primarily reached parents with comparatively well-off socioeconomic backgrounds and was unable to engage parents with lower socioeconomic status. The composition of our sample aligns with other evaluations of parenting programs. In the digital Triple T program, 56% of participants had a university degree, and 70% felt at least reasonably financially comfortable, despite efforts to include disadvantaged families (Baker et al., 2017). Similarly, in another study evaluating the digital Triple P program, 55.7% had a university degree (Franke et al., 2020). This is in line with research showing that e-mental health offers are used less often in groups with low socioeconomic status and might further increase health disparities between different socioeconomic groups (Standaar et al., 2025). In addition, parents with a low socioeconomic status are more likely to drop out of parental training programs (Chacko et al., 2016) and tend to benefit less than parents with a higher socioeconomic status (Lundahl et al., 2006). This is particularly relevant given that, lower socioeconomic status has been associated with higher levels of parental stress which in turn is associated with an increased risk for child abuse (Martins et al., 2023). This emphasizes the importance of developing accessible e-mental health interventions for parents with lower socioeconomic status.

A scoping review examining recruitment strategies found that, across studies on e-mental health interventions targeting populations with low socioeconomic status, approaches such as personal or telephone contact, face-to-face outreach, and referrals through general practitioners were more successful than recruitment based on leaflets or internet-based approaches (Al-Dhahir et al., 2022). The use of social media channels as well as especially, the distribution of leaflets and posters in relevant spaces were central recruitment strategies in this study. We also contacted psychotherapists offices and clinics in our region as well as health professionals in our clinic to increase the rate of referral by health professionals, but only few participants were reached through these channels. Barriers such as limited time, workflow constraints, and organizational factors were reported to restrict referrals to additional preventive services in hospital care in a study on the implementation of digital parent training in primary care (Fehrenbacher et al., 2020), and these factors were likely also relevant in the present context. In future studies, establishing collaborations with psychotherapist, pediatricians and general practitioners as well as more face-to-face outreach seems crucial to reach more socioeconomically disadvantaged parents.

Satisfaction with the preliminary game prototype was considerably lower compared to previous studies evaluating internet-based parental trainings. For instance, an internet-based behavioral parent training program for children with ADHD achieved a mean intervention satisfaction score of 28.02 (SD = 2.87) (Fan et al., 2025). However, the low satisfaction rating in this study needs to be viewed with caution, given that the prototype was still preliminary and lacked extensive content, design features, personalization, and gamification elements. In line with this, open-ended feedback primarily highlighted the very limited available parent-child interactions, the underrepresentation of certain child age groups, and the need for more response options, as well as improved visual elements and reduced text. Improvements to these key elements are expected to increase participant satisfaction in future studies, as the overall concept was well received, including the lighthouse metaphors and videos, the parent-centered focus, the selection of challenging parent-child interactions, and the use of multiple response options per situation. Moreover, the SUS score observed in this study, indicating good usability, is comparable to findings from other studies, such as for a mobile app for parents of children with ADHD, which reported a SUS score of 80.35 (SD = 11.16) (Păsărelu et al., 2023). Ensuring usability throughout the formative developmental process is important (Kip et al., 2022), as this provides now a solid foundation to further refine the game prototype. However, as an important feasibility finding, 26.9% of the recruited participants were unable to complete the registration process and therefore did not initiate the intervention, indicating a relevant implementation barrier. Moreover, on a descriptive level, differences in depressive and somatization symptoms between initiators and non-initiators were observable despite not reaching statistical significance. Therefore, the registration process might have been a particularly strong barrier for participants experiencing greater symptom burden. The registration process should therefore be revised to simplify the procedure before conducting a subsequent evaluation study.

To explore naturalistic usage patterns among parents, the prototype did not include predefined recommendations for frequency, duration, or timing of use, allowing parents to engage with the prototype according to their own preferences. There is a considerable divergence between self-reported usage time and frequency of the game prototype and the average estimates recorded via Firebase, indicating a possible overestimation in self-reported data due to social desirability effects or recall bias. However, data security restrictions in Firebase prevented access to individual usage data, so only a limited number of aggregated indicators could be analyzed, rather than individual usage patterns. As the tested preliminary prototype contained only two parent-child interactions, with not all age groups of children represented, the observed average usage time of 11 min (excluding the time spent watching the LPP videos) seems consistent with these limitations. The aim of the authors is to further develop the prototype by including a variety of different child-parent interactions for different age group between 0- and 14 years. Usage recommendations for parents in a full version of the game could be aligned with the *Happy for Families* game, in which parents were expected to work through a total of 30 family conflict scenarios, with a suggested pace of approximately two scenarios per week over a period of 15 weeks (Priego-Ojeda et al., 2025). However, the interviews conducted with interested parents during the co-design process showed that the game-based approach may not fully address the needs of all parents (Braun et al., n.d.). The parents in this sample expressed concerns about feeling evaluated as a parent or being directly compared to other parents and expressed a preference for direct access to psychoeducational content and exercises (Braun et al., n.d.). To address these concerns, the focus in the further developmental process could be shifted toward the use of selected gamification strategies in an app-based application, rather than developing a full game using game-based scenarios.

To select appropriate measures for inclusion in a full RCT, we included exploratory measures evaluating perceived stress, mental burden, mentalizing and parent-child relationship quality. The perceived stress and depression scale both provided a preliminary indication for a possible pre-post improvement and warrants investigation in a subsequently adequately powered study. However, no indication for improvement was observed regarding mentalizing skills or parent-child relationship quality. Baseline scores indicate that the participants in this study had on average mild levels of depressive symptoms and moderate stress levels. The PSI sum score was elevated for parents (M = 72.92) and their children (M = 57.46), but below the levels reported in a clinical sample of parents (M = 81.82) and their children (M = 68.75), who participated in an inpatient parent-child treatment program (Fritz et al., 2017). Average PSS-10 sum scores (M = 19.19) were comparable to a German community sample of mothers with children aged 10–79 months (M = 19.37) indicating similar levels of perceived stress (Wildner et al., 2024). Mentalizing skills at baseline based on the PRFQ were also comparable to the average mentalizing scores attained in a German community sample of mothers with young children (Wildner et al., 2024) and showed no substantial impairment. As such, ceiling effects may be present, particularly with regard to mentalizing ability. Thus, this proof-of-concept study was conducted in a predominantly healthy, well-functioning sample of parents experiencing relatively normative stress levels and adapting to challenging parent–child situations, who nevertheless showed a strong interest in improving their parenting skills. As a preliminary prototype was tested within this co-design process, this study represents a first step in assessing its potential and risks in a functional sample. The next step will be to evaluate a further developed version of the prototype in a parental sample characterized by impaired mentalizing abilities and greater parent–child relationship difficulties.

### 4.1 Limitations and strengths

The study has several limitations. *First*, as a methodical limitation, no causal inferences can be made as no control group was included. The reported clinical outcomes are highly exploratory due to the limited statistical power, since the sample size was only powered to address feasibility problems. The observed pretest-posttest effects could also be attributed to spontaneous remission, simultaneous treatments, selection-, or attrition bias (Armijo-Olivo et al., 2022). *Second*, even though most estimates were in the acceptable range, internal consistencies varied across measures and assessment points. In particular, the low estimate for the Interest and Curiosity subscale (ω_t0_ = 0.54) of the PRFQ warrant cautious interpretation. Also, some reliability estimates of the DERS-SF and MEK were relatively low. Given the small sample size, reliability estimates may be imprecise and unstable (Padilla and Divers, 2015). *Third*, due to the formative character of the prototype, a priori criteria or progression thresholds for the feasibility indicators were not predefined. Thus, the feasibility findings provide preliminary descriptive information on recruitment, acceptability, usability, intervention initiation and engagement, but do not allow for a formal assessment against predefined feasibility criteria and progression thresholds. *Fourth*, the external validity and generalizability of the results is limited, as approximately half of the study sample also participated in the previous co-design study and was not naïve regarding the underlying theoretical concepts and mock-up design as the average user. Furthermore, negative effects of the co-design process such as disappointment or frustration are possible (Slattery et al., 2020), as the user feedback could only in part be incorporated before the start of the low-fidelity feasibility testing. Moreover, the relatively low PSI scores suggest, that we were not able to recruit parents with particular high level of parental stress. Thus, the results cannot be generalized to burdened parents experiencing high parental stress levels. In line, the representativeness of the study sample is constrained to female, well-situated parents with high educational level while parents from socially disadvantaged backgrounds were underrepresented. As this was a first formative feasibility study, future evaluations should use more targeted inclusion criteria to reach parents experiencing elevated levels of parental stress. The intervention could be targeted specifically to parents with a defined PSI cut-off or to parents with children that experience behavioral, emotional or cognitive difficulties. Recruitment could be more targeted to communities experiencing socioeconomic disadvantages. *Fifth*, due to the early developmental stage and preliminary character of the low-fidelity prototype, content, graphic design, gamification elements and personalization options were very limited which led, as indicated by the open feedback questions, to a drop in the satisfaction rating. However, the formative evaluation in this developmental stage enabled to ensure early-on that sufficient usability was provided which can be built upon for the further development process.

This study is also characterized by two notable strengths. *First*, the mobile game prototype was developed in a participative co-design process by conducting interviews and focus groups with interested parents regarding the content, design and gamification elements to be included. The advantage is a better fit between user needs and system requirements, resulting in higher user satisfaction and effectiveness (Antonini, 2021). Specifically, in the design of digital interventions for individuals with severe mental illness, co-designed approaches are expected to enhance effectiveness and adoption, while reducing power imbalances and strengthening self-esteem and self-efficacy (Schouten et al., 2022). *Second*, the formative evaluation of this low-fidelity prototype allows for early identification and remediation of potential issues before the conduct of a resource-intensive full RCT.

## 5 Conclusions

This study provides a proof-of-concept for an innovative mentalization-based mobile game prototype grounded in the LPP, co-designed with interested parents to reduce parental burden. The feasibility of the currently preliminary low-fidelity prototype is limited, as reflected in low satisfaction ratings. However, revisions to content, design, and gamification elements based on user feedback from the co-design process are expected to enhance intervention satisfaction. The results indicate a within-group reduction in depressive symptoms and stress at post-intervention, although changes were small, inconsistent and cannot yet be attributed to the intervention. The clinical effectiveness of a high-fidelity version of this mobile game prototype has yet to be established and needs to be investigated in further studies. Overall, these findings show, that more research is needed to establish whether such interventions can improve parental burden and family functioning, with potential implication for the prevention of child maltreatment.

## Declaration of Generative AI and AI-assisted technologies in the writing process

During the preparation of this work, the authors used ChatGPT (OpenAI) in order to improve the language and readability of selected paragraphs. The scientific content, interpretations, and conclusions are solely those of the authors. After using this tool, the authors carefully reviewed and edited the text and take full responsibility for the content of the published article.

## Funding

This project was supported by the IMPETUS project (https://impetus4cs.eu/). IMPETUS is funded by the European Union's Horizon Europe research and innovation program under grant agreement number 101058677. Views and opinions expressed are, however, those of the authors only and do not necessarily reflect those of the European Union or the European Research Executive Agency (REA). Neither the European Union nor the granting authority can be held responsible. IMPETUS had no role in the design of the study and the writing of the manuscript.

## Declaration of competing interest

The authors declare that they have no known competing financial interests or personal relationships that could have appeared to influence the work reported in this paper.
