# Supplementary material for: Proof-of-concept of a co-designed mobile game prototype to enhance parental mentalizing: Results from a single-group pretest-posttest feasibility trial (HelpingParentsMinds)
Source: Internet Interv. 2026 Sep 29;46:101003. doi: 10.1016/j.invent.2026.101003 (PMC13654488; doi:10.1016/j.invent.2026.101003)
Supplement: Supplementary file 1 [file mmc1.docx]

**Appendix A. System Usability Scale (SUS)**

**Figure A.1**

*SUS rating scale according to Blattgerste et al.* (2022)

*Note.* dashed middle line represents mean SUS score, continuous middle line represents median.

Available at: <https://sus.mixality.de/>

**Appendix B. Correlations matrix**

**Table B.1**

*Spearman (rho) correlations for feasibility outcomes and psychological burden, stress and symptoms at baseline*

| Variable | 1 | 2 | 3 | 4 | 5 | 6 | 7 | 8 | 9 | 10 |
| --- | --- | --- | --- | --- | --- | --- | --- | --- | --- | --- |
| 1. Duration of use | 1 |  |  |  |  |  |  |  |  |  |
| 2. Frequency of use | 0.59* | 1 |  |  |  |  |  |  |  |  |
| 3. CSQ-I | 0.49* | 0.33 | 1 |  |  |  |  |  |  |  |
| 4. SUS | 0.11 | 0.02 | 0.54* | 1 |  |  |  |  |  |  |
| 5. Psychological burden ^a^ | -0.11 | 0.12 | -0.03 | 0.03 | 1 |  |  |  |  |  |
| 6. PSI | -0.07 | -0.20 | -0.06 | -0.22 | 0.68** | 1 |  |  |  |  |
| 7. PSS-10 | -0.08 | 0.22 | -0.30 | -0.26 | 0.69** | -0.60** | 1 |  |  |  |
| 8. PHQ-9 | -0.49* | -0.09 | -0.28 | -0.06 | 0.72** | -0.60** | 0.66** | 1 |  |  |
| 9. PHQ-15 | -0.13 | 0.28 | 0.15 | -0.09 | 0.55** | -0.42* | 0.29 | 0.47* | 1 |  |
| 10. PHQ-stress | -0.17 | 0.21 | 0.02 | 0.04 | 0.73** | -0.60* | 0.62** | 0.62** | 0.74** | 1 |

*Note.* *indicates *p* < .05, ** indicates *p* < .01; low: ρ = .10, moderate: ρ = .30, high: ρ = .50; CSQ-I = Client Satisfaction with Internet Interventions, SUS = System Usability scale, PSI = Parental Stress Index, PSS-10 = Perceived Stress Scale, PHQ = Patient Health Questionnaire

^a^ Self-reported parental burden: 1 = not burdened at all to 10 = very heavily burdened

Appendix C. Open-ended feedback

Table C.1

*Open-ended positive feedback from the participants*

| Topic | Quotes | *N* (%) |
| --- | --- | --- |
| Usability | “Ux/Ui has not crashed. Easy to operate”  “Simple language”  “Comprehensible structure”  “It was kept very simple”  “Overall, I liked it a lot. Easy to use and gives a pleasant feeling.”  “Structuring, clarity […]”  “Structure, language, […]” | 7 (36.8) |
| Lighthouse metaphors and videos | “Lighthouse as a symbol of parenthood”  “I like the metaphor of the lighthouse for the change of perspective.”  “I think the lighthouse concept is great and I think it's good that it was also integrated with the videos. After each situation, there were short info boxes explaining the metaphors without going into unnecessary detail.”  “Explanatory videos linked […]”  “[…], videos, […]”  “[…], forwarding to explanatory videos” | 6 (31.6) |
| Focus on parent | “I also liked the fact that I was important as a parent, as the focus was also placed on my own feelings and I was encouraged to self-regulate.”  “The fact that many different topics were discussed in detail and that we were asked a lot about how we felt.”  “You become more mindful” | 3 (15.8) |
| Situation selection | “I liked the opportunity to choose from different situations”  “The situations shown” | 2 (10.5) |
| Response options | “Many possible answers.”  “The answer options take the user by the hand and get you involved in the game. It's also good that you get suggestions if you choose a bad answer.” | 2 (10.5) |
| Mix of education and application | “I thought it was good that, on the one hand, general background knowledge was provided, on the other hand, there was the opportunity to try out situations (by acting them out) and, finally, the option was offered to be accompanied through a situation of your own.”  “I also liked the fact that - in my opinion - a lot of background knowledge went into the development of the prototype, it seemed very well-founded to me.” | 2 (10.5) |

*Note. N* (%) participants reporting positive aspects within the respective category at post-assessment (*N* = 19).

Table C.2

*Open-ended negative feedback from the participants*

| Topic | Quotes | *N* (%) |
| --- | --- | --- |
| Expanding range (Situations, responses) | “Of course, I would have appreciated a larger selection of situations, but I didn't expect anything else, as it was announced at the start of the course that only a few situations would be available. I particularly missed a situation relating to toddlerhood, mainly typical tantrums (you have peeled the banana ‘wrong’ and your child starts to scream).”  “More situations in the prototype (too short).”  “Unfortunately, my children were not in the category for which the two sample cases were created.”  “Unfortunately, the age range that includes my children was missing.”  “Difficult, as the two existing examples were not helpful for me.”  “The error message “not yet implemented” appeared several times when I tried to test a situation”  “Should offer several options for correct behavior for different personalities”  “The two scenarios were not suitable for me. That's why it was difficult to use the app correctly”  “It didn't really match my current situation either. “  “It would be good if more game variants were offered.”  “Situations didn't fit my children and everyday situations, it wasn't very helpful and is difficult to evaluate.” | 11 (57.9) |
| Visualization | “Texts too long”  “Less text.”  “More videos and pictures for illustration”  “I found some of the texts too long. The app design could be a bit more visually appealing.”  “Graphically it could be more appealing. Animations etc.” | 5 (26.3) |
| Embedding videos in the prototype | “If there was a way to play the video directly in the app, that would have made it much easier for me to use this point.”  “Integrate video in the game (no new window). Play video exactly where it is about (not e.g. from minute 2:44)” | 2 (10.5) |
| Gamification | “For me, it's not a game but rather a question-and-answer simulation.”  “Much more interactive […] It should be more playful and less multiple choice.” | 2 (10.5) |
| Level of difficulty | “The answers are predictable. You know what you have to answer. Something simple. The child doesn't react in the same way as in the situations.”  “The answers were pretty clear to me, what I should choose. And therefore quite transparent” | 2 (10.5) |
| Clear structuring | “On the other hand, I would have liked to be able to choose from three options immediately after selecting the situation: whether I want to read the background information at the beginning (or skip it because I already know it), whether I want to play through the situation in the app, or whether I want to be guided directly through my own situation.”  “The different steps within a situation could have been presented more clearly. After going back a few times, I lost track of which step I was currently in. Perhaps a small field could be used to show which step of the situation you are currently in, e.g. with the help of the mentalization steps.” | 2 (10.5) |
| Mindfulness exercises | “On the one hand, I would have liked not only to be instructed to calm down, but possibly also to be guided through a short exercise (2-minute breathing pause or similar), as experience has shown that it is sometimes difficult to regulate myself effectively in heated situations.”  “When annoyed, it's difficult to react appropriately and keep your feelings down.” | 2 (10.5) |
| Adaptation to the user | “What I would also have loved would have been a slightly more individualized response to my input, instead of the same old informational text at the point where I could enter my feelings and assumptions about my child's feelings. (E.g. I say I'm angry and then get a text that focuses on the feeling of anger in particular, possibly directly linked to strategies for self-regulation).” | 1 (5.3) |

*Note. N* (%) participants reporting negative aspects within the respective category at post-assessment (*N* = 19).
